# Effect of Home-Based High-Intensity Interval Training in Patients With Lacunar Stroke: A Randomized Controlled Trial

**DOI:** 10.3389/fneur.2019.00664

**Published:** 2019-06-28

**Authors:** Rikke Steen Krawcyk, Anders Vinther, Nicolas Caesar Petersen, Jens Faber, Helle K. Iversen, Thomas Christensen, Kate Lykke Lambertsen, Shazia Rehman, Tobias Wirenfeldt Klausen, Egill Rostrup, Christina Kruuse

**Affiliations:** ^1^Department of Physiotherapy and Occupational Therapy, Herlev Gentofte Hospital, University of Copenhagen, Copenhagen, Denmark; ^2^Neurovascular Research Unit, Department of Neurology, Herlev Gentofte Hospital, University of Copenhagen, Copenhagen, Denmark; ^3^QD-Research Unit, Herlev Gentofte Hospital, University of Copenhagen, Copenhagen, Denmark; ^4^Center for Translational Neuromedicine, University of Copenhagen, Copenhagen, Denmark; ^5^Division of Endocrinology, Department of Internal Medicine, Faculty of Health and Medical Sciences, Herlev Gentofte Hospital, University of Copenhagen, Copenhagen, Denmark; ^6^Department of Neurology, Stroke Center Rigshospitalet, University of Copenhagen, Copenhagen, Denmark; ^7^Department of Neurology, Nordsjællands Hospital, University of Copenhagen, Copenhagen, Denmark; ^8^Department of Neurobiology Research, Institute of Molecular Medicine, University of Southern Denmark, Odense, Denmark; ^9^Department of Neurology, Odense University Hospital, Odense, Denmark; ^10^BRIDGE–Brain Research Interdisciplinary Guided Excellence, Department of Clinical Research, University of Southern Denmark, Odense, Denmark; ^11^Department of Radiology, Herlev Gentofte Hospital, University of Copenhagen, Copenhagen, Denmark; ^12^Department of Haematology, Herlev Gentofte Hospital, University of Copenhagen, Copenhagen, Denmark; ^13^Mental Health Center Glostrup, Copenhagen, Denmark

**Keywords:** aerobic exercise, high-intensity interval training, home-based physical activity, secondary stroke prevention, lacunar stroke

## Abstract

**Background:** High-intensity interval training (HIIT) is superior to moderate-intensity continuous training in improving cardiorespiratory fitness in patients with cardiovascular disease, but is it safe, feasible and effective in patients with stroke? We investigated feasibility and effect of early, home-based HIIT in patients with lacunar stroke combined with usual care vs. usual care, only.

**Methods:** Patients with minor stroke (severity: 55/58 point on the Scandinavian Stroke Scale) were randomized to HIIT or usual care in a randomized, controlled trial. We measured the following outcomes at baseline and post-intervention: cardiorespiratory fitness monitored as power output from the Graded Cycling Test with Talk Test (GCT-TT; primary outcome), physical activity, fatigue, depression, well-being, stress, cognition, endothelial function, blood pressure, body mass index, and biomarkers.

**Results:** We included 71 patients (mean age 63.7 ± 9.2), 49 men, 31 in intervention group. Home-based HIIT was feasible with no reported adverse events in relation to the intervention. No significant change between the groups in GCT-TT power output was detected (*p* = 0.90). The change in time spent on vigorous-intensity activity was 2 h/week and 0.6 h/week, intervention and usual care, respectively (*p* = 0.045). There were no significant differences between groups in the remaining secondary outcomes.

**Conclusion:** HIIT was feasible and safe in patients with lacunar stroke. Patients can engage early in home-based HIIT when involved in choosing exercise modality and guided by weekly motivational phone calls. Within 3 months, HIIT did, however, not yield effect on cardiorespiratory fitness. We await further evaluation of long-term effects of this intervention on continued regular physical exercise and cardiovascular event.

**Clinical Trial Registration:** https://clinicaltrials.gov, identifier NCT02731235

## Introduction

Stroke is one of the leading causes of mortality and disability (1). Approximately 90% of strokes are attributed to modifiable risk factors, and 75% of the global stroke burden may be avoided by control of behavioral and metabolic risk factors (2). In stroke survivors, lifestyle modifications combined with preventive medication are recommended to be initiated early, as there is a 3.7–6.7% risk of recurrence within the first 90 days after stroke onset (3, 4). The international multicentre study INTERSTROKE (5) highlighted ten common modifiable risk factors associated with ischemic stroke and identified physical inactivity as one of the most important. Several studies have shown that physical activity has a protective effect against stroke (6, 7), as physical activity has the potential to improve cardiorespiratory fitness and reduce blood pressure, lipids, and body weight, thus improving cardiovascular health (8, 9). Physical activity and stroke risk factors can evoke changes in inflammatory, endothelial and cardiovascular biomarkers, which may be used to monitor risk factor load, disease progression, or effect of specific interventions (10).

Increasing the physical activity, including increasing the frequency, volume, and intensity of exercise, is associated with health benefits (11) and decreased risk of a future stroke in healthy individuals (12–14). Whether this effect also applies to risk of a recurrent stroke in patients with a manifest cerebrovascular event has yet to be fully established (15).

Following stroke, a low level of physical activity (16) and aerobic exercise (17) is often reported, perhaps due to specific barriers to physical activity after stroke. The most frequently reported barriers are environmental barriers, such as challenges in ambulation and transport to the required training facilities, and personal barriers, including lack of motivation and knowledge on how to initiate and maintain an exercise program. Some patients also indicate a fear of recurrent stroke with increased exercise (18, 19). A poorer outcome of very early, within 24-h of stroke onset, initiated intensive mobilization has pushed this concern further forward (20).

High-intensity interval training (HIIT) is an exercise modality that could be feasible in patients with stroke due to the low time commitment involved. HIIT has been shown to be a powerful alternative to moderate-intensity, continuous training (MICE) to improve cardiorespiratory fitness in cardiac rehabilitation (21). In addition, improved endothelial function after HIIT compared with MICE has also been reported based on flow-mediated dilation of the brachial artery in patients with cardiovascular disease (22). Based on these studies, we hypothesized that a HIIT programme designed to overcome the typical barriers for physical activity in stroke patients is feasible and could improve cardiorespiratory fitness, participation in physical activity, endothelial function, and quality of life in patients with lacunar stroke. We aimed to investigate the effect of early initiated HIIT for 12 weeks post-stroke in addition to usual care compared with the effects of usual care only.

## Methods

### Study Design

We designed a randomized controlled trial with a parallel-group design. Patients were randomized at a 1:1 ratio to either intervention or usual care, within 3 weeks of a stroke. Following randomization, patients were followed for 3 months. The reporting of this study adheres to the CONSORT statement (23).

### Ethical Approval

The trial was approved by The Danish Data Protection Agency (ID: HGH-2015-021) and the Research Ethics Committee in the Capital Region of Denmark (Trial Registration number: H-15012371). Eligible patients provided written informed consent prior to study participation. Furthermore, the study was registered at URL: ClinicalTrials.gov (ID: NCT02731235, registered January 2016).

### Recruitment

Patients were recruited from January 2016 until January 2018 from the stroke unit at hospitals in the Capital Region of Copenhagen, Denmark; Herlev Gentofte Hospital, Rigshospitalet Glostrup, and Nordsjællands Hospital. All assessments were carried out at the stroke unit at Herlev Gentofte Hospital. During the recruitment period, medical records were screened daily by the study coordinator to identify patients with lacunar stroke. Eligible patients were enrolled consecutively within 1–21 days of stroke onset.

### Participants

Patients 18 years or older diagnosed with a first-time lacunar stroke or a recurrent event of lacunar stroke were enrolled in the study. A lacunar stroke was defined according to the Trial of Org 10172 in Acute Stroke Treatment (TOAST-criteria) (24). This definition included patients with clinical symptoms with a verified relevant brain stem or subcortical hemispheric lesion (<2 cm in diameter in the acute phase) based on computed tomography (CT) scan or magnetic resonance imaging (MRI) scan (25). Additionally, the patients had a severity of neurological symptoms, categorized as “mild” on the Scandinavian Stroke Scale (SSS) (43–58 points) (26). Patients had to speak and read Danish and provide informed consent. We excluded patients with previous large-artery stroke, unstable cardiac condition, atrial fibrillation, pacemaker, uncontrolled hypertension, uncontrolled diabetes, artery stenosis >50 %, symptoms or comorbidities not allowing exercise on a stationary bicycle, dyspnoea caused by heart or pulmonary disease, aphasia, or dementia that interfered with understanding the protocol and physical examinations.

### Procedures

During hospital admission all patients had routine examination for stroke cause and risk factors. These tests included a chest x-ray, 48-h cardiac event monitoring (Novacor, Rueil Malmaison, France), carotid artery imaging (ultrasound), and routine blood tests (full blood count, glucose, electrolytes, lipids, creatinine, etc.). Furthermore, an MRI (sequences: diffusion-weighted imaging, the derived apparent diffusion coefficient value, fluid attenuated inversion recovery, and T2^*^-weighted images on a 1.5 T clinical system using an 8-channel standard head coil) (Achieva, Philips Healthcare, Best, The Netherlands) was acquired to confirm diagnosis, localization, and presence of acute and old ischemic lesions and microbleeds.

All patients complied with medication in accordance with their stroke physicians' recommendation during admission. In addition, all patients were offered usual occupational therapy and physiotherapy during their hospital stay when needed. As part of the usual care procedure, patients were also offered a follow-up visit at the outpatient clinic.

During the trial, we made three study amendments to increase the recruitment rate: (1) we extended the study to also include patients with acute transient ischemic attack (TIA) and concomitant MRI-verified signs of a previous lacunar stroke (5 patients) (previously only patients with acute/subacute stroke were included), (2) we included patients within 21 days after onset of symptoms (previously only included patients within 7 days), and (3) we expanded the number of recruitments sites to three stroke units in the Capital Region of Copenhagen (previously only one stroke unit). Amendment 1 and 2 were made 6 months after trial initiation (June 28th, 2016) and amendment 3 was made 18 months after trial initiation (June 27th, 2017).

### Randomization and Blinding

After completing all assessments at baseline, the patients were randomized into one of two groups: usual care and exercise intervention or usual care only. The randomization procedure was based on equal allocation with randomly varying block size. The block-randomization was computer-generated (8 blocks of 10, mixed with 5 blocks of 4) and carried out by a research assistant not involved in the study. Sealed opaque envelopes were made by the research assistant, stored, and administrated by health personnel not involved in the study. The outcome assessor, data analysts, and study coordinator were all blinded to the randomization process. Immediately following baseline assessments, the study coordinator collected the next envelope from the health personnel. The consecutively enrolled patient opened the envelope and was allocated to either intervention group or usual care group.

### Intervention

#### Both Intervention and Usual Care Group

At baseline, all patients attended a motivational talk with the study coordinator to encourage lifestyle changes, and they were introduced to an exercise catalog with a range of suggestions for modes of aerobic exercise (e.g., brisk walking stair stepping, stationary bicycling, outdoor cycling, running, indoor rowing, high knee exercises, and swimming).

#### Intervention Group

In addition to usual care, the intervention group performed home-based HIIT daily 3 × 3 min with 2 min of active recovery, 5 days per week for 12 weeks (27) (Figure S1). HIIT was defined as exercise at 77–93% of the maximum heart rate, corresponding to 14–16 on the Borg-rated perceived-exertion scale (28) or “not able to speak comfortably” on the Talk Test (29–31). The Talk Test determined the initial intensity of the intervention, and the patients progressed the work load and the cadence as they improved throughout the exercise program. In each session, the patients were encouraged to reach an exercise intensity at which they were no longer able to speak comfortably. To determine their speaking comfort the intervention group carried a pocket-sized, laminated standardized text passage (cue card), which became redundant as they got familiar with the text. They also wore a stop watch to time the exercise intervals. The exercise modality was self-chosen assuming it was performed at high intensity. Patients in the intervention group were provided with a stationary bicycle if required (Kilberry® Magnetic Bike JC-950, Proteus Sports Inc., Linkou Township, Taiwan) for use at home to ensure an easily accessible exercise modality. Before initiating the exercise program, the study coordinator visited each patient at home to introduce the exercise program, including the use of the Talk Test. Furthermore, the exercise sessions were tracked by the patients in an exercise diary to encourage adherence to the exercise program and to report any adverse events.

For further motivation and control of completion of work-out, the study coordinator contacted the patients during the exercise period, on a weekly basis to ensure compliance, identify equipment malfunction, and to register any adverse events (AE). An AE was defined as any untoward and unintended response during the exercise intervention with serious adverse event (SAE) or without hospital admission, which did not necessarily have a causal relationship to the intervention. We registered (S)AE from start of the intervention, throughout the intervention period weekly until 2 weeks after cessation of the intervention. Events of definite TIA or stroke were considered outcome measures (32).

#### Usual Care Group

The usual care group received secondary preventive medication and advice on self-managed lifestyle changes. Furthermore, the usual care group was asked to resume their habitual level of physical activity and to track their physical activity in an exercise diary.

### Outcome Measures

All outcome measures were obtained at baseline and post-intervention (3 months after initiation of the intervention) (Figure S2).

#### Cardiorespiratory Fitness

The primary outcome, the Graded Cycling Test with Talk Test (GCT-TT), measured sub-maximal cardiorespiratory fitness monitored as power output in Watts (W). GCT-TT was performed on a stationary bicycle (Monark 928E-G3, Vansbro, Sweden) and identified the exercise intensity at which the patient perceived that it was no longer possible to speak comfortably due to excessive breathing. The workload was increased by 15W every minute and each minute the patient also recited a standardized text passage (33). When the patient was no longer able to speak comfortably the test terminated. A detailed test protocol has previously been published (34) establishing the feasibility and measurement error for groups [12.9W; standard error of measurement (SEM_95_)] and for individuals with lacunar stroke [18.3W; smallest real difference (SRD)] (34). The SRD corresponded to two steps (30W) in the GCT protocol, which represents a change for an individual patient (34).

#### Post-stroke Fatigue

Post-stroke fatigue was measured by the Multidimensional Fatigue Inventory (MFI-20), a generic self-report instrument covering five domains of fatigue: general fatigue, physical fatigue, reduced activity, reduced motivation, and mental fatigue (35). We used a cut-off score ≥12 points in the general fatigue-score as a measure of overall fatigue, as proposed in the original development of the scale (35). Higher score indicated higher degree of fatigue (35).

#### Depression

Depression severity was assessed by the Major Depression Inventory (MDI). The questionnaire consisted of 12 questions on mood-related symptoms during the previous 2 weeks, with responses provided on a 6-point Likert scale. The total score ranked from 0–50 points. A high score indicated more severe depression (36), a score >20 points indicated mild depression, and a score between 15 and 20 points was interpreted as incipient depression (37).

#### Mental Well-Being

Mental well-being was measured by the generic World Health Organization-Five Well-being Index (WHO-5) questionnaire (38). WHO-5 included five positive statements with responses given on a 6-point Likert scale, which corresponded to the patient's own experience for the previous 2 weeks. The total score ranked from 0–100 points and 50 points indicated reduced well-being or long-term stress (39). In prior studies, the Danish population showed an average score of 69 points (38).

#### Chronic Stress

Chronic stress was evaluated using an algometer (Ull-Meter®, Ull Care, Hellerup, Denmark) with corresponding recording of pain threshold on the sternum, expressed as pressure pain sensitivity (PPS) (40, 41). With the patient in a supine position, the most sensitive point on the sternum was identified, and the hand-held algometer was applied to the sternum with gradually increasing intensity until the pain threshold was reached. The algometer automatically transformed the pain threshold into a logarithmic scale of sensitivity from 30–100 PPS units, with a cut-off ≥60 correlating with markers of a stress syndrome (42). The reading on the algometer was blinded to the observer until completion of the assessment (40, 41).

#### Cognition

We screened for mild cognitive impairments using the Montreal Cognitive Assessment (MoCA), including the following nine cognitive domains: attention, concentration, executive functions, memory, language, visuospatial ability, conceptual thinking, calculations, and orientation. The total score ranked from 0–30 points, where a score ≥26 points was considered normal cognition (43). MoCA was previously shown to be valid and reliable in patients with lacunar stroke or white matter lesions (44).

#### Endothelial Function and Arterial Stiffness

Endothelial function was assessed by digital plethysmography to determine the peripheral arterial tonometry using EndoPAT2000 (Itamar Medical Ltd., Caesarea, Israel) and registered as the reactive hyperaemia index (RHI). An RHI score >1.67 was recommended as a cut-off for normal endothelial function in the user manual of the EndoPAT2000 device.

Arterial stiffness was registered as the augmentation index (AI), together with the heart-rate-corrected AI at a heart rate of 75 beats per min (AI@75). Lower scores indicate greater elasticity of the arteries. Endothelial function and arterial stiffness were both calculated using the EndoPAT software package version 3.4.4. All measures were conducted in accordance with conditions described by the manufacturer, and a detailed procedure has previously been described (45).

#### Blood Pressure

Baseline blood pressure was measured at each visit after an overnight fast, following 5 min of rest with the patient in a supine position using an automatic blood pressure monitor (Microlife® BP A100/ Microlife® BP A3L Comfort, Widnau, Switzerland). We aimed for a blood pressure <130/90 mmHg as recommended for patients with a recent lacunar stroke or TIA (46).

#### Biomarkers

Venous blood was drawn to assess biomarkers associated with cardiovascular and endothelial function, and inflammation. The cardiovascular biomarkers (pro-adrenomedullin [Pro-ADM], pro-atrial natriuretic peptide [Pro-ANP], and copeptin) are biomarkers to regulate the vascular tone and blood pressure (47, 48). The inflammatory biomarkers (interleukin-6 [IL-6] and tumor necrosis factor [TNF]), and endothelial biomarkers (intercellular adhesion molecule-1 [ICAM-1], vascular cell adhesion molecule-1 [VCAM-1], vascular endothelial growth factor [VEGF], and E-selectin) supplemented the endothelial function data retrieved from EndoPAT. Blood was centrifuged at 4,000 rpm for 15 min at 4°C within 45 min after sampling and all samples were stored at −80°C until analysis. Inflammatory biomarkers and endothelial biomarkers were analyzed using commercially available kits from Mesoscale, Rockville, USA (V-PLEX Plus human: IL-6 kit, TNF kit, ICAM-1 kit, VCAM-1 kit, VEGF kit, and E-selectin kit) according to the manufacturer's instructions. Samples were analyzed in duplicates. Prior to measurement, the samples were diluted two-fold in Diluent 41, and MSD Discovery Workbench software was used for analysis (49). The cardiovascular biomarkers were analyzed using commercially available kits and software from BRAHMS GmbH Hennigsdorf, Germany (KRYPTOR compact PLUS human: Pro-ADM kit, Pro-ANP kit, and copeptin kit) according to the manufacturer's instructions. Fasting plasma insulin concentration (50) was determined using a commercially available kit (ELISA kit, DRG Instruments GmbH, Marburg, Germany) according to the manufacturer's instructions.

#### Body Mass Index

Body mass index (BMI) was calculated based on height as measured in centimeters and body weight as measured in kilograms (body weight/height^2^) using a body composition monitor (OMRON HBF-500-E; Kyoto, Japan). Obesity was defined by a BMI ≥30 kg/m^2^ and was associated with increased risk of cardiovascular disease and first-time stroke (46).

#### Physical Activity

We evaluated physical activity both subjectively using the self-reported questionnaire Physical Activity Scale version 2.1 (PAS2) (51) and objectively using an accelerometer (AX3, Axivity, York, UK). In PAS2, patients reported their average physical activity behavior 2 weeks prior to hospital admission (baseline) and 2 weeks prior to the post-intervention assessment. PAS2 revealed daily time spent on sleep, sitting down at work, standing/walking at work, heavy physical work during working hours, active commuting to/from work, and sedentary behavior, including television watching and reading. Additionally, in PAS2, patients recorded the weekly time spent on light-intensity, moderate-intensity, and vigorous-intensity activity during leisure time (51). Each activity corresponded to a specific MET (metabolic equivalent of task) intensity according to the Compendium of Physical Activity (52), which allows physical activity to be calculated as a total 24-h MET score. To estimate the average duration of activity per day, each score from the three leisure-time activities was divided by seven. Total time reported per day spent on the activities was calculated by adding the hours spent on all activities. If the total time reported was below or above 24 h, we added or subtracted time that was not accounted for to the category “light activity,” as suggested in a previous study (51).

Objective physical activity was acquired by a wireless three-axis accelerometer (AX3, Axivity, York, UK) fixed with double-sided adhesive tape (VIP Tape, Skinlock International, Charleroi, Belgium) anteriorly on the right medial thigh with a water-resistant patch (Fixomull® transparent, BSN Medical, Inc., Hamburg, Germany). The AX3 was programmed to record activity patterns for 8 days and 7 nights with a frequency of 25 Hz, using manufacturer's software (Open Movement v.1.0.0.28). After download, data were analyzed with Acti4, a custom-made script in MATLAB (version: R2013a), including a previous described algorithm (53) to identify everyday physical activity types, such as walking, running, cycling, walking stairs, standing up, and sitting down.

#### Other Data Collection

The following data were collected from the patients or from patient records: age, sex, clinical symptoms at time of hospital admission, age, type and location of the lesion, mobility, family status, occupation, education level, pre-existing diabetes, hypertension and hypercholesterolemia upon hospital admission, and smoking and drinking habits.

### Statistical Analyses

#### Sample Size

Sample size calculation was based on the primary outcome (GCT-TT power output). Using a two-tailed 5% level of significance and a power of 80%, to detect a minimal clinical important average difference of 23W, a sample size of 84 patients (42 in each group) was needed. With allowance for a dropout rate of 15%, we aimed to enroll 100 patients in total.

No prior studies have established a minimal clinical important difference for the GCT-TT power output. The decision to choose a 23W average difference was based on a previous study in cardiac patients (54) reporting that the smallest detectable average change for a group of patients (SEM_95_) was 18.3W and the smallest real difference for an individual patient (SRD) was 25.9W (corresponding to two 15W-steps in the incremental test protocol). An average difference for a group of patients clearly exceeding 23W are closer to two incremental steps (30W) than to one (15W) incremental step in the test protocol. Thus, it was our best estimate of a minimal clinical important average difference between groups.

#### Analysis

We analyzed complete outcome data according to the group the patients were randomized to, regardless of patient compliance. All available data for each patient were included in the analysis. Missing data were not imputed. Data on demographics and baseline characteristics were compared between the groups using independent *t*-test for comparison of means and Fisher's exact test for comparison of categorical variables.

To evaluate outcome changes between the groups for both the primary outcome and for secondary outcomes, we used analysis of covariance (ANCOVA) for continuous variables, the Mann-Whitney test for ordinal variables (estimating the difference at the post-intervention assessment), and Fisher's exact test for categorical variables. For evaluation of differences within each treatment group for both primary and secondary outcomes, we used a paired *t*-test for continuous variables and a Wilcoxon signed rank test for ordinal variables. The differences, both between groups and within groups, were calculated and given as mean difference with 95% confidence intervals (CI). Before analysis, all variables were tested for normal distribution using histograms and QQ-plots, and if necessary, the data were logarithmically transformed. All data were analyzed with a two-tailed test and with a statistical significance level set at *p* < 0.05. Data were analyzed using Microsoft Excel 2010 (Microsoft Corporation, Redmond, WA, USA) and IBM SPSS Statistics 22 (Armonk, NY, USA). Statistical planning and analysis were conducted in cooperation with one of the authors, a biostatistician (TWK).

## Results

No significant differences were found between the groups in terms of demographics or baseline characteristics (Table 1), and no adverse events related to the intervention were recorded during the study.

**Table 1:** Patient characteristics at baseline.

**Variable**	**Intervention (*n* = 31)**	**Usual care (*n* = 32)**
Men, *n* (%)	23 (74)	26 (81)
Age, years (mean ± SD)	63.7 ± 8.9	63.7 ± 9.2
**Mobility**		
Without walking aids, *n* (%)	28 (90)	30 (94)
Pre-stroke use of walking aids, *n* (%)	0	1 (3)
Scandinavian stroke scale, points (mean ± SD)	54.6 ± 5.8	55.3 ± 4.4
**Family status**		
Cohabitants, *n* (%)	22 (71)	23 (72)
Living alone, *n* (%)	9 (29)	9 (28)
**Education**		
Primary education, *n* (%)	1 (3)	3 (9)
Apprenticeship, *n* (%)	9 (29)	9 (28)
Upper secondary education/high school, *n* (%)	1 (3)	1 (3)
Short-cycle tertiary education, *n* (%)	6 (19)	2 (6)
Bachelor or equivalent, *n* (%)	9 (29)	5 (16)
Masters, equivalent or higher, *n* (%)	5 (16)	12 (38)
**Lesion, age**		
First-time lacunar stroke, *n* (%)	18 (58)	17 (53)
Recurrent lacunar stroke, *n* (%)	3 (10)	2 (6)
Only older lacunar infarct verified on MRI, combined with clinical symptoms corresponding with a TIA, *n* (%)	1 (3)	4 (13)
First-time lacunar stroke but also sequela lacunar stroke verified on MRI, *n* (%)	9 (29)	9 (28)
Acute/subacute infarct, *n* (%)	30 (97)	28 (88)
Previous clinical symptoms, *n* (%)	8 (26)	4 (13)
Thrombolysis, *n* (%)	**3 (10)**	**3 (9)**
**Hemispheric localization of lesion**		
Right hemisphere, *n* (%)	19 (61)	18 (56)
Left hemisphere, *n* (%)	12 (39)	12 (38)
Bilateral, *n* (%)	0	2 (6)
**Lesion localization**		
Thalamus	9	5
Basal ganglia	17	19
Frontal lobe	2	2
Pons	1	4
Medulla oblongata	1	1
Corpus callosum	0	1
Motor cortex	1	0
**Clinical symptoms on admission**		
Paresis/dexterity of extremities, *n* (%)	23 (74)	18 (56)
Sensory impairments of the extremities, *n* (%)	11 (35)	12 (38)
Facial palsy, *n* (%)	7 (23)	11 (34)
Dysarthria, *n* (%)	6 (19)	11 (34)
Vertigo, *n* (%)	5 (16)	8 (25)
Visual problems, *n* (%)	5 (16)	2 (6)
**Cardiovascular risk factors**		
Hypertension at hospitalization, *n* (%)	26 (84)	25 (78)
Hypertension previously known, *n* (%)	17 (55)	14 (44)
Pre-existing diabetes, *n* (%)	3 (10)	2 (6)
BMI, kg/m^2^ (mean ± SD)	28 ± 5	26 ± 4
**Smoking**		
Current smokers, *n* (%)	6 (19)	6 (19)
Previous smokers, *n* (%)	14 (45)	16 (50)
Non-smokers, *n* (%)	11 (36)	10 (31)
Alcohol consumption^**^		
< Health authorities' recommendations, *n* (%)	18 (58)	21 (66)
>Health authorities' recommendations, *n* (%)	13 (42)	11 (34)
**Lipids**		
Total cholesterol, mmol/L (mean ± SD)	5.6 ± 1.3	5.5 ± 1.4
LDL, mmol/L (mean ± SD)	3.3 ± 1.3	3.1 ± 1.0^♣^
HDL, mmol/L (mean ± SD)	1.4 ± 0.5	1.4 ± 0.4
Triglycerides, mmol/L (mean ± SD)	2.0 ± 1.1	1.7 ± 0.8^♣^
**Blood pressure**		
Systolic pressure, mmHg (mean ± SD)	149 ± 22	147 ± 21
Diastolic pressure, mmHg (mean ± SD)	85 ± 10	89 ± 11

*No significant difference was detected between the groups*.

** *The Danish Health authority recommends <7 units per week for women (1 unit equals 1 glass of wine) and <14 units per week for men (55)*.

♣ *n = 30*.

### Study Recruitment

We screened records on all patients admitted to the stroke unit (*n* = 3,098) daily and excluded 2,969 patients who did not have a diagnosis of lacunar stroke. A total of 58 patients with lacunar stroke declined participation due to the following reasons: reduced mental surplus (the patients were too tired or excused themselves with no energy, as seen with mental fatigue) (*n* = 21), no reason given (*n* = 25), pain (*n* = 8), and work obligations (*n* = 4). Of the declining patients, 31 were men (mean age 70 ± 9 years) and 27 women (mean age 69 ± 11 years). In total, 71 patients with lacunar stroke were included with a mean symptom severity score (SSS score) on admission of 55 ± 5 points. The patients were recruited 1–17 (mean 6 ± 4) days after stroke onset and the first assessment visit was performed 12 ± 7 days after hospital admission.

**Figure 1:**
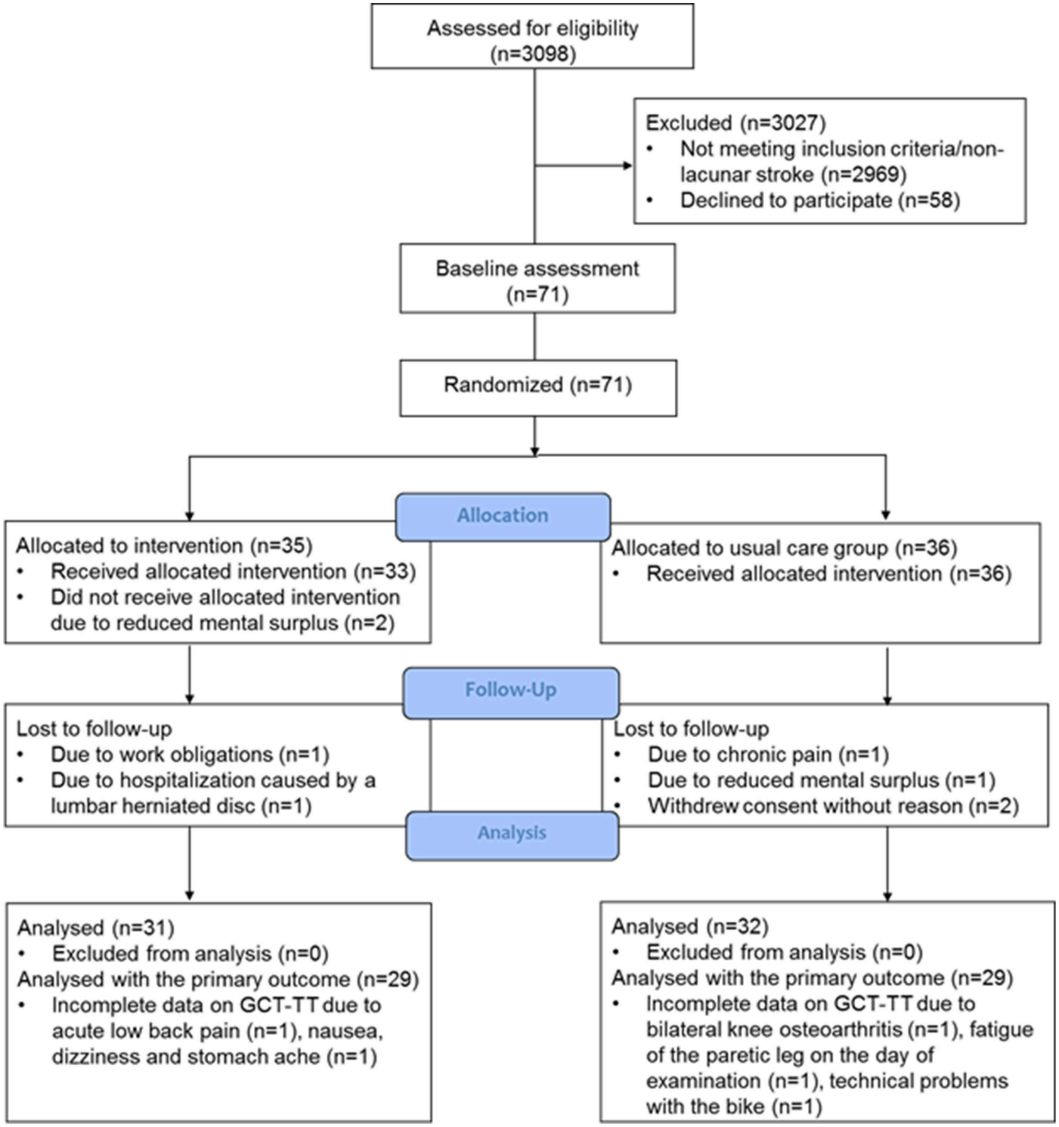
Flow diagram of the randomized controlled trial to investigate effect of home-based HIIT in patients with lacunar stroke.

Between March 2016 and April 2018, 63 patients attended the post-intervention assessment. Eight patients (11%), four in each group, were lost to the post-intervention assessment. The cause of drop-out in the intervention group were: one patient had adverse event unrelated to the study intervention (a lumbar herniated disc allegedly induced by heavy lifting during a house renovation project), one patient had work obligations, and two patients reported reduced mental surplus. In the usual care group, the cause of drop-out were: one patient had chronic pain in her knee from osteoarthritis and was awaiting surgery, one patient complained of reduced mental surplus, and the remaining gave no cause (Figure 1). The patients lost to the post-intervention assessment had a mean age of 63 ± 11 years, five were women, and six lived alone. Of the 63 patients included in the analysis, five patients (8%) were readmitted to the hospital within the first 3 months of lacunar stroke. All readmissions were identified as serious adverse events, however unrelated to the intervention (one patient from the intervention group and four from the usual care group). Two patients were identified to have a clinical diagnosis of a new TIA, one patient was admitted for chest pain, and two patients for dizziness and malaise.

The study was terminated as planned, but due to low recruitment, we did not fulfill the intended inclusion number, and extension of inclusion was not possible due to time and finances.

### Primary Outcome

In total, 58 patients were analyzed with the GCT-TT at the post-intervention assessment. Three patients from the usual care group did not complete the GCT-TT, one had fatigue of the paretic leg at baseline, prior to any intervention, one had longstanding knee osteoarthritis and refused to perform the GCT-TT at the post-intervention assessment for the fear of worsening the pain, and one patient did not complete due to technical problems with the stationary bicycle. Two patients from the intervention group did not complete the GCT-TT: one because of nausea, dizziness, and stomach ache on the day of the post-intervention assessment considered not to be related to the intervention. The fourth patient experienced acute lower back pain at the day of post-intervention assessment and was not able to sit on the stationary bicycle (Figure 1).

There was no significant difference over time between groups in GCT-TT power output (*p* = 0.90; Table 2). However, 17 of 29 patients in the intervention group improved their GCT-TT power output from baseline to post-intervention assessment, and 11 of 29 patients in the usual care group improved their GCT-TT power output from baseline to the post-intervention assessment (*p* > 0.05; data not shown). Eight patients in each group improved ≥30W in GCT-TT power output, and this change corresponded to two steps in the GCT-TT protocol, equivalent to a clinically relevant change.

**Table 2:** Results for outcomes measured at baseline and at the post-intervention assessment for both groups.

**Variable**	**Intervention**		**Usual care**		**Difference between groups from baseline to post-intervention assessment**		
	**Baseline**	**Post-interventi on assessment**	**Baseline**	**Post-interventi on assessment**	**Difference in change**	**95% CI**	p^*^
	**(*n* = 31)**	**(*n* = 31)**	**(*n* = 32)**	**(*n* = 32)**			
**Primary outcome**							
^♣^GCT-TT, W, (mean ± SD)	118.5 ± 43.1	126.2 ± 46.3	119.5 ± 44.0	126.2 ± 47.9	0.90	−13.9–15.7	0.90
**Secondary outcomes**							
Post-stroke fatigue, points (mean ± SD)	10 ± 5	11 ± 5	11 ± 3	10 ± 4	1.3	−0.4–3.1	0.13
^▴^Chronic stress, points (mean ± SD)	62 ± 16	59 ± 15	57 ± 16	55 ± 17	0.7	−5.7–7.1	0.83
Depression, median [IQR]	5 [1;10]	6 [3;13]	9 [4;12]	7 [4;15]			0.86^†^
Mental well-being, points (mean ± SD)	65 ± 23	69 ± 16	64 ± 18	69 ± 17^$^	−0.6	−7.7–6.5	0.86
Cognition, median [IQR]	27 [27;29]	29 [28;30]^$^	28 [26;30]	29 [28;30]^$^			0.37^†^
Physical activity (PAS2)							
- Physical activity, MET (mean ± SD)	39.7 ± 4.7	39.1 ± 4.8	39.3 ± 4.8	40.5 ± 4.2	−1.6	−3.6–0.3	0.10
- Sleep, hours/week (mean ± SD)	52.4 ± 6.7	55.1 ± 8.3	51.6 ± 9.6	52.1 ± 8.6	2.4	−0.7–5.5	0.10
- Sedentary behavior, hours/week (mean ± SD)	40.7 ± 15.3	42.1 ± 17.9	44.1 ± 18.2	39.6 ± 17.3^$^	5.1	−1.1–11.3	0.10
- Light activity, hours/week (mean ± SD)	66.6 ± 16.8	62.3 ± 19.1	63.9 ± 19.2	66.8 ± 19.5	−6.2	−14.1–1.8	0.13
- Moderate activity, hours/week (median [IQR])	6.2 [2.6;10.0]	6.0 [2.0;10.0]	5.5 [2.0;8.9]	6.9 [4.4;10.5]			0.28^†^
- Vigorous activity, hours/week (median [IQR])	0.0 [0.0;2.0]	2.0 [0.0;3.0]^$^	0.0 [0.0;2,4]	0.6 [0.0;2.0]			0.045^†^
^ᴥ^Endothelial function							
- RHI, index (mean ± SD)	2.6 ± 1.0	2.6 ± 0.8	2.3 ± 0.5	2.3 ± 0.5	0.1	−0.2–0.4	0.4
Arterial stiffness							
- AI (% change in peak systolic pulse wave) (median [IQR])	20 [10;34]	24 [5;39]	11 [3;34]	14 [4;34]			0.43^†^
- AI@75 (% change in peak systolic pulse wave) (median [IQR])	12 [3;25]	19 [−1;25]	2 [−3;29]	7 [−3;27]			0.45^†^
Blood pressure							
- Systolic, mmHg (mean ± SD)	149 ± 22	144 ± 18	147 ± 21	141 ± 16^$^	2.5	−4.9–9.7	0.5
- Diastolic, mmHg (mean ± SD)	85 ± 10	83 ± 10	89 ± 11	84 ± 7^$^	0.5	−3.2–4.1	0.8
BMI, kg/m^2^ (mean ± SD)	27.5 ± 4.5	27.4 ± 4.3	25.6 ± 3.6	25.4 ± 3.6^$^	0.3	−0.1–0.6	0.2
^**^Objective physical activity (hours/day)							
- Activity (including cycling, climbing stairs, running, and walking), (median [IQR])	0.08 [0;0.38]	0.05 [0;0.5]	0.08 [0;0.66]	0.06 [0;0.6]			0.92^†^
- Stand/move around, (median [IQR])	1.40 [0.91;2.69]	1.58 [0.89;2.73]	1.63 [1.07;2.79]	1.67 [1;2.69]			0.78^†^
- Sedentary behavior (including sitting/lying), (median [IQR])	19.0 [16.9;20.2]	18.9 [17.2;20.6]	18.4 [17.3;19.9]	18.5 [17.1;19.7]			0.62^†^
- Total steps/day (mean ± SD)	7,208 ± 4,170	7,068 ± 3,953	8,251 ± 2,814	7,877 ± 2,163			0.8

*Difference in change is only provided when a mean difference could be calculated*.

*IQR, interquartile range*,

* *calculated by ANCOVA, calculated by Mann-Whitney test (between the groups at the post-intervention assessment)*,

♣ *29 patients in each group*,

$ *data from within-group analysis are significant*,

** *26 patients in each group*,

ᴥ *30 patients in each group*,

▴ *31 patients in the usual care group*.

### Secondary Outcomes

#### Physical Activity

At baseline, 21 patients in the intervention group and 18 patients in the usual care group reported that they did not spend time on vigorous-intensity activity. We found a significant behavioral change in time spent on vigorous-intensity activity (PAS2) in the intervention group compared with the usual care group from baseline to the post-intervention assessment (*p* = 0.045; Table 2).

**Figure 2:**
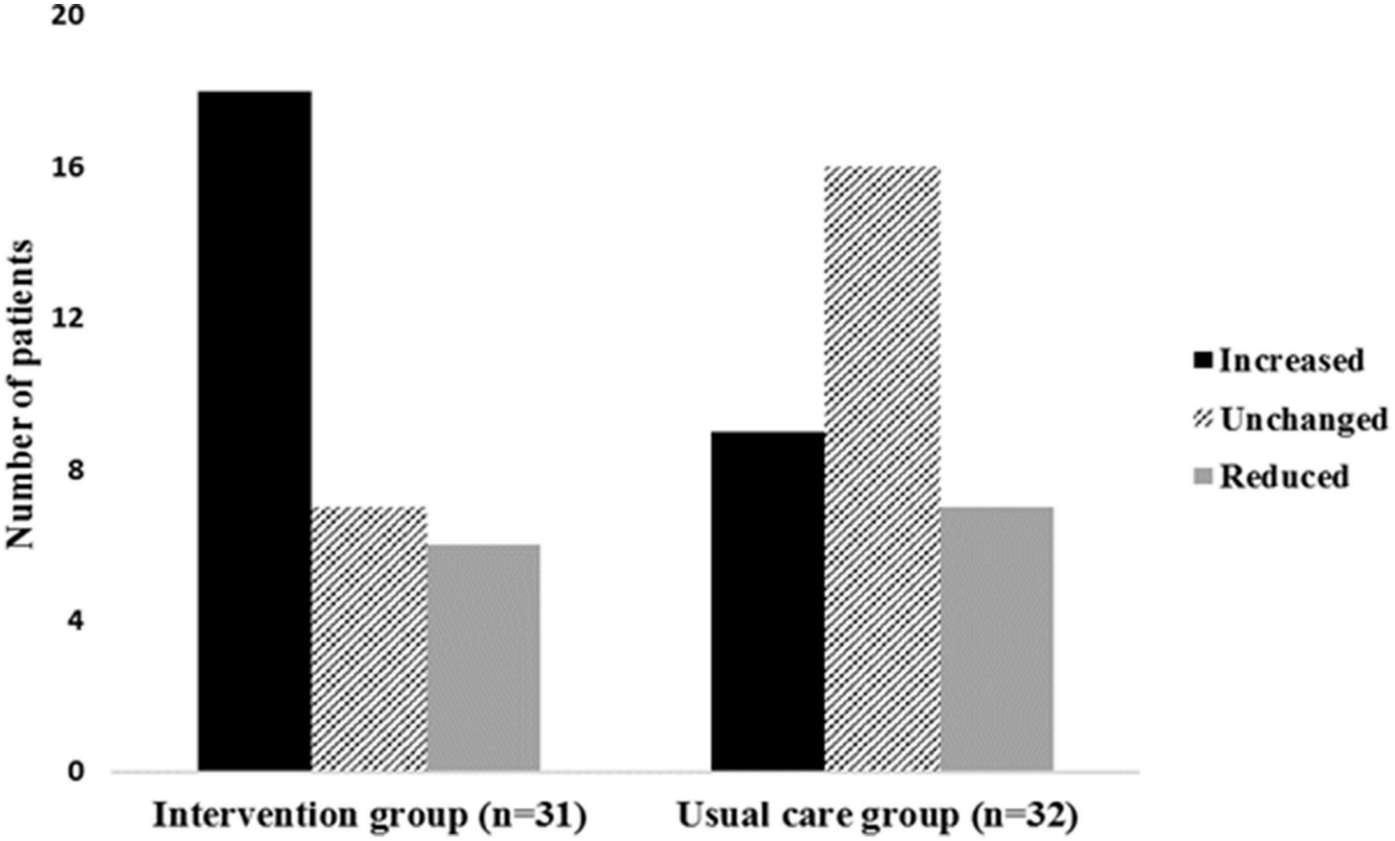
Change in vigorous-intensity activity from baseline to the post-intervention assessment in both groups. The figure shows the number of patients in each group who either increased, did not change or reduced their time spent on vigorous-intensity activity from baseline to post-intervention assessment.

In the intervention group, 18 patients increased (58%), 6 patients decreased (19%), and 7 patients (23%) did not change their participation in vigorous-intensity activity. In the usual care group 9 patients (28%) increased, 7 patients decreased (22%), and 16 patients (50%) did not change their participation in vigorous-intensity activity (Figure 2). A higher proportion of patients in the intervention group increased their participation in vigorous-intensity activity from baseline to the post-intervention assessment (*p* = 0.01).

**Figure 3:**
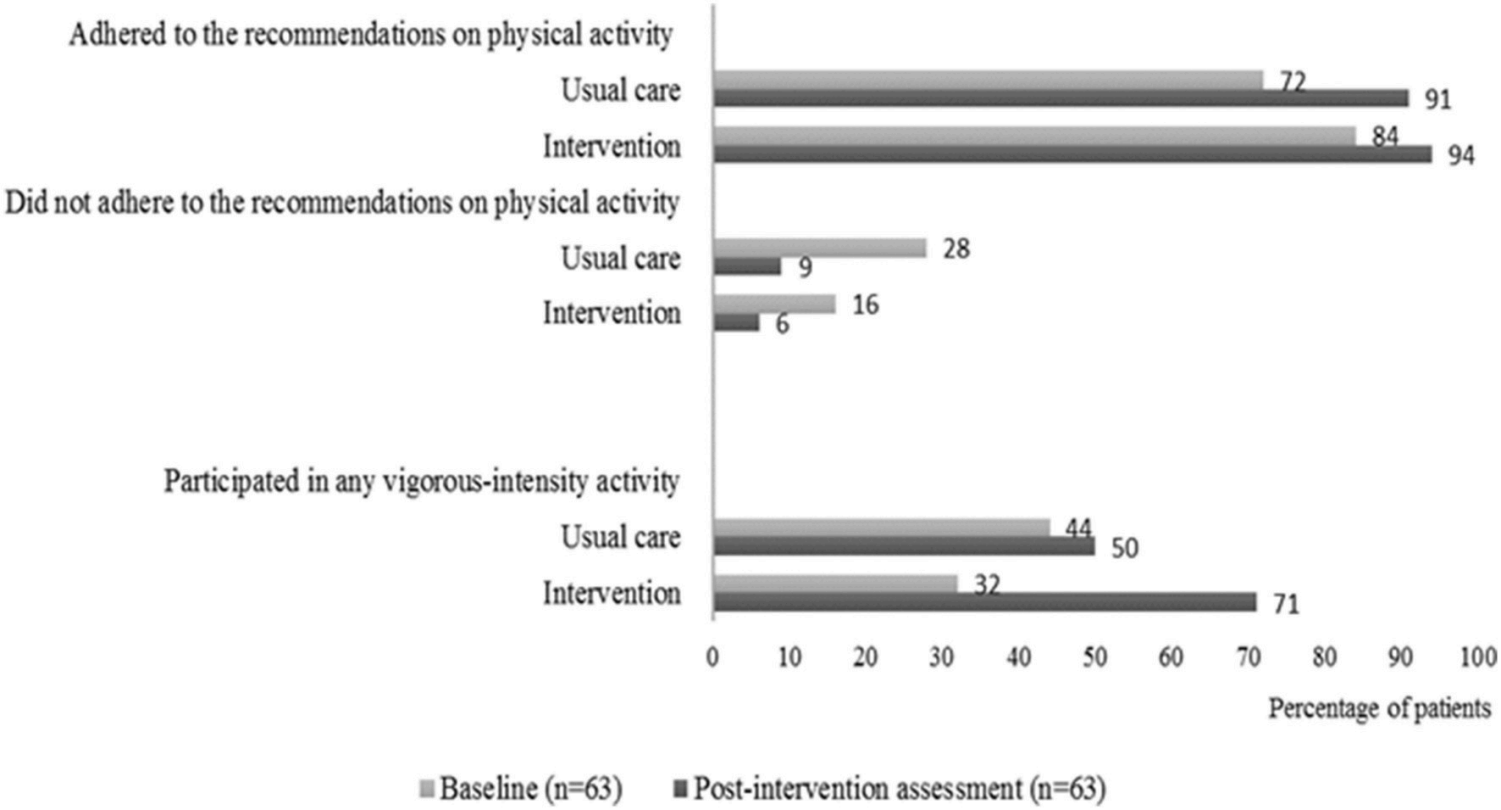
Self-reported adherence to physical activity. Percentages of patients in the intervention group and in the usual care group who adhered or did not adhere to the international recommendations on physical activity at baseline and at the post-intervention assessment. The patients were considered adherent if they performed vigorous-intensity activity (≥75 min per week), moderate-intensity activity (≥150 min per week), or an equivalent combination. The figure also shows the percentage of patients in the intervention group and in the usual care group who participated in any vigorous-intensity activity at baseline and at the post-intervention assessment.

In the intervention group, 15 of 21 patients (71%) and 5 of 18 patients in the usual care group (28%) went from, not participating in vigorous-intensity activity at baseline to participate in any vigorous-intensity activity at the post-intervention assessment. Furthermore, we found that more patients both from the intervention group and the usual care group performed more physical exercise at the post-intervention assessment compared to baseline. The most substantial positive change was seen for the intervention group regarding their increase in vigorous-intensity activity (Figure 3).

#### General Well-Being and Cardiovascular Function

No change was detected between groups in the level of post-stroke fatigue, chronic stress, depression, mental well-being, cognition, blood pressure or endothelial function (Table 2).

#### Biomarkers

Statistically significant reductions in lipids were observed within both groups (*p* = 0.00, *p* = 0.00, respectively) (Table 3). Fasting insulin was reduced in both groups over time, however the decrease in the usual care group was marginally reduced compared to the intervention group (*p* = 0.048). A significant reduction in ICAM-1 in the usual care group compared to the intervention group (*p* = 0.006) and VCAM-1 was slightly increased in the intervention group compared to the usual care group (*p* = 0.024) (Table 3). The changes were, however, small with no consistent effect on specific functions (inflammation, endothelium and cardiovascular).

**Table 3:** The results for biomarkers measured at baseline and at the post-intervention assessment for both groups.

	**Intervention**		**Usual care**		**Difference between groups from baseline to post-intervention assessment**		
**Variable**	**Baseline**	**Post-intervention assessment**	**Baseline**	**Post-intervention assessment**	**Difference in change**	**95% CI**	p^*^
	**(*n* = 31)**	**(*n* = 31)**	**(*n* = 32)**	**(*n* = 32)**			
**Lipids**							
- Total cholesterol, mmol/L (mean ± SD)	5.6 ± 1.3	4.2 ± 0.9^$^	5.5 ± 1.4	4.1 ± 1,2^$^	0.05	-0.4–0.5	0.8
- LDL, mmol/L (mean ± SD)	3.3 ± 1.3	2.1 ± 0.6^$^	3.1 ± 1.0^ᴥ^	2.1 ± 0.8^ᴥ^*^$^*	−0.03	-0.4–0.3	0.9
- HDL, mmol/L (mean ± SD)	1.4 ± 0.5	1.5 ± 0.5	1.4 ± 0.4	1.4 ± 0.4	0.10	0.00–0.28	0.3
- Triglycerides, mmol/L (mean ± SD)	2.1 ± 1.1	1.3 ± 0.6^$^	1.7 ± 0.8^ᴥ^	1.2 ± 0.7^ᴥ^*^$^*	−0.06	-0.03–0.2	0.6
**Blood samples**							
*Cardiovascular biomarkers*							
- Pro-adrenomedullin, nmol/L (median [IQR])	0.57 [0.53;0.71]	0.62 [0.54;0.75]^$^	0.58 [0.52;0.64]	0.54 [0.54;0.68]			0.58^†^
- Pro-atrial natriuretic peptide, pmol/L (median [IQR])	64 [50;94]	72 [57;105]^$^	71 [51,84]	73 [50;93]			0.40^†^
- Copeptin, pmol/L (median [IQR])	6.5 [4.6;10.2]	6.7 [5.5;10.4]	6.2 [4.6;9.5]	6.7 [4.8;9.7]			0.55^†^
- Insulin pmol/L (median [IQR])	117 [82;166]^▴^	106 [82;148]^▴^	91 [77;138]^ᴥ^	87 [65;103] ^ᴥ^*^$^*			0.048^†^
*Inflammatory biomarkers*							
- IL-6 pg/ml (median [IQR])	1.40 [0.81;3.12]	1.13 [0.82;1.73]^$^	0.83 [0.71;1.17]	1.10 [0.75;1.52]			0.35^†^
- TNF pg/ml (median [IQR])	2.27 [1.88;3.53]	2.48 [1.97;2,74]	2.21 [1.89;2.81]	2.32 [1.86;3.20]			0.43^†^
*Endothelial biomarkers*							
- ICAM-1 μg/mL (median [IQR])	0.80 [0.64;0.95]	0.78 [0.62;1.26]^*$*^	0.82 [0.59;1.08]	0.68 [0.55;0.82]^$^			0.006^†^
- VCAM-1 μg/mL (median [IQR])	1.11 [0.60;1.34]	1.16 [0.77;1.52]^*$*^	1.06 [0.63;1.46]	0.98 [0.61;1.24]			0.024^†^
- VEGF pg/ml (median [IQR])	29.8 [25.1;41.7]^♠^	30.3 [22.5;42.7]^♠^	31.2 [25.2;41.2]^♢^	29.8 [21.1;42.8]^♢^			0.99^†^
- E-selectin, ng/mL (median [IQR])	6.22 [4.12;9.88]	5.54 [4.61;7.48]	5.09 [3.60;7.19]	4.76 [4.07;6.58]			0.34^†^

*Difference in change is only provided when a mean difference could be calculated*.

*IQR, interquartile range*,

* *calculated by ANCOVA*,

† *calculated by Mann-Whitney test (between the groups at the post-intervention assessment)*,

$ *data from within-group analysis are significant*,

♢ *n = 25*,

▴ *n = 28*,

♠ *n = 29*,

ᴥ *n = 30*.

### Exercise Adherence

According to the exercise diaries, patients in the intervention group reported that they, on average, performed the exercise program 56 of 60 days (93%). For reference, 60 days of exercise corresponds to 5 days per week for 12 weeks. In the intervention group, 10 of 31 patients (32%) performed the exercise program more than 5 days per week (>100% adherence), while 24 patients (77%) in the intervention group performed the exercise program ≥4 days per week (≥80% adherence).

A total of 23 patients (74%) in the intervention group chose to complete the aerobic exercise sessions on the provided stationary bicycle, whereas the remaining 8 patients performed brisk walking (1 patient), stair stepping in combination with outdoor cycling on different days (2 patients), running (2 patients), brisk walking combined with outdoor cycling on different days (1 patient), brisk walking combined with rehabilitation twice a week in the community (1 patient), and indoor rowing (1 patient).

According to the patients' exercise diaries, in which they recorded the number of days with any kind of activity during the 12-week study (84 days), the intervention group reported that they were physically active 64 ± 12 days (5.4 days per week), whereas the usual care group reported physical activity on 51 ± 24 days (4.2 days per week). Physical activity, measured objectively with accelerometers, did not show a significant difference within or between the groups from baseline to the post-intervention assessment (Table 2).

## Discussion

The main findings of this randomized controlled trial on home-based HIIT were that an early initiated HIIT exercise program was feasible and safe in patients who have had a minor stroke (lacunar stroke), and that a significant proportion of patients in the intervention group markedly increased their time spent on vigorous-intensity activity compared to patients in the usual care group. This reported increase in physical activity, however, was not translated into an improvement in GCT-TT power output. HIIT did not significantly improve the general well-being (depression, chronic stress, post-stroke fatigue, cognition, and quality of life), cardiovascular function (blood pressure and endothelial function). For biomarker outcomes (cardiovascular, inflammatory, and endothelial) the results were ambiguous with no obvious beneficial effect of HIIT on the endothelial response.

Only a few studies have investigated the effect of HIIT in stroke patients (56), and only three studies were randomized controlled trials (57–59). In the present study, we have investigated HIIT as a home-based intervention with distant supervision for patients with lacunar stroke. HIIT is more often performed as individual treadmill training after stroke to improve mobility, gait speed, and gait stride (56). Only one randomized controlled trial reported a superior effect of HIIT vs. MICE to improve cardiorespiratory fitness in patients with stroke (57). More studies that evaluate the effects of the various available exercise methods are needed to confirm these findings. The current study highlights the need for additional research to investigate the effect of HIIT on cardiorespiratory fitness in stroke patients.

The exercise intensity was not monitored during the home-based training and too low exercise intensity may explain part of the missing cardiovascular effects. We chose not to measure the exercise intensity with heart rate monitors based on clinical observations in a small test of feasibility which showed that the patients with lacunar stroke were not able to comply with the available monitoring equipment. Their familiarity with, and ability to use smartphones was not consistent, and some patients developed a skin rash following use of electrocardiography electrodes for more than 3–5 days. Furthermore, the commercially available heart rate monitors at the initiation of the study did not have the required memory capacity or battery life for weeklong monitoring.

Another cause of discrepancy between reported activity and lack of cardiovascular effects may be the relatively short exercise duration (15 min of exercise 5 days a week). When planning the study, our hypothesis was that patients with lacunar stroke were likely to be unfamiliar with physical activity and feasibility of the intervention was a main priority. There is no general consensus on the ideal HIIT protocol for motor recovery and improved cardiorespiratory fitness after stroke (60). The most frequently used HIIT protocol in patients with cardiovascular disease targeting endothelial function, was 4 × 4 min, three times per week (22). Additionally, adherence to the international recommendations on physical activity for health, require 75 min of weekly vigorous-intensity activity (61), thus we chose 15 min 5 times a week.

Furthermore, the current home-based HIIT intervention was designed to be easy for patients to perform at home, easy to translate into clinical practice, and at low-cost in an effort to reduce the known barriers for physical activity in stroke survivors (18, 19).

All patients, also those in the usual care group, were aware of being enrolled in an exercise study with regular assessments, and all patients were informed on the importance of physical activity following stroke. These features of the study may have encouraged all patients to be physically active. In addition, the supervision of all patients by a physiotherapist during the baseline assessment of the GCT-TT may have decreased the fear of doing aerobic exercise following stroke also in the usual care group. Despite encouragements and a subtle increase in physical activity, a change exceeding the previously established SEM_95_ of 12.9W (34) was not observed either within the groups or between the groups in GCT-TT power output.

Patients with an ischemic stroke or a transient ischemic attack (TIA) have an increased risk of recurrent stroke and cardiovascular events (62). Consequently, medical intervention or change in lifestyle factors are often suggested. Secondary prevention using medication is effective in preventing recurrent stroke (46). However, the evidence exploring the effect of lifestyle changes on recurrent stroke is not solid (46). Two recent reviews explored lifestyle interventions, including cardiovascular exercise to prevent cardiovascular events after stroke and TIA (63). They found no effect on cardiovascular events, but a significant reduction in systolic blood pressure, fasting insulin and fasting glucose, and an increase in high-density lipoprotein cholesterol (63, 64). However, our study was not able to confirm similar significant findings on cardiovascular function.

A priori we were curious to see if we could detect an improvement in general well-being (depression, chronic stress, post-stroke fatigue, cognitive function, and mental well-being) in favor of the intervention group. We did, however not detect a significant improvement between the groups within the first 3 months of stroke. The few previous studies reporting on the efficacy of exercise (aerobic exercise and resistance training) on depression and well-being showed inconsistent results (65), though a trend toward exercise having a positive impact on cognitive function has been suggested (65). There is not yet sufficient literature available on the efficacy of non-pharmacological interventions to prevent or treat post-stroke fatigue (66), likewise there is a lack of knowledge on the effect of exercise on stress, none of these outcomes were affected in this study, however, the baseline values were surprisingly good leaving little room for possible improvement. Of note, the sample size was small, and the effects of HIIT may become more consistent and significant in a larger sample size.

Studies have showed that HIIT had a protective effect on endothelial function in patients with cardiovascular disease (22). Also, aerobic exercise reduces the blood pressure in patients with stroke or TIA (67). Consequently, we expected an improvement on endothelial function in favor of the intervention group, but such was not detected within the 3 months of exercise. It may be related to a potentially low exercise intensity and to a short daily exercise duration but may also be caused by a low incidence of or too well-managed co-morbidities to decrease endothelial function such as diabetes or hypertension in our patient population.

Based on the literature investigating patients with cardiovascular disease, we primarily expected a decrease in biomarkers following physical exercise. Inflammatory biomarkers (IL-6 and TNF) were shown to decrease in patients with coronary heart disease (10, 68). Endothelial biomarkers, ICAM-1, VCAM-1 (68, 69), VEGF (70, 71) were all shown to decrease in patients with cardiovascular risk factors, whereas there was insufficient evidence on the efficacy of exercise on E-selectin. Cardiovascular biomarkers (pro-ADM, pro-ANP) were shown to decrease in patients with heart failure (72, 73), and limited literature is available on the efficacy of exercise on Copeptin. We found only minor changes in the assessed biomarkers without clinical relevance between the groups. When comparing the baseline values from the included stroke patients to values from healthy individuals (manufacturer's normative values), the levels of inflammatory, cardiovascular biomarkers and ICAM-1 were generally higher in the stroke patients. This difference may reflect the underlying cardiovascular risk factors in our patient sample.

During the study period, we found an 8% rate of hospital readmittance, of which two patients (3%) had a confirmed clinical diagnosis of TIA. In comparison, previous studies have identified a risk of 3.7–6.7% of stroke recurrence within the first 90 days after stroke onset (3, 4). This is interesting, and it could be speculated that patients with minor stroke may be less predisposed to stroke recurrence compared to patients with larger stroke severity. Prospective studies with large sample sizes are, however, needed to further elucidate this.

By providing the patients with a stationary bicycle, contacting them on a weekly basis and monitoring them at baseline, we succeeded in engaging patients with lacunar stroke into being more physically active, including performing vigorous-intensity activity. These findings are very encouraging compared to the results of a large international multicentre study (ExStroke), which found no effect of repeated encouragement and verbal instructions on physical activity in patients with ischemic stroke (74). In addition, the Look AHEAD study (75) did not show an effect on cardiovascular events when aiming for lifestyle changes. The study investigated weight reduction by eating fewer calories combined with increased physical activity (unsupervised home-based exercise) by using repeated encouragement (individual sessions and group meeting) in patients with type 2 diabetes. In Look AHEAD, half of the study population dropped out or had no change in physical activity. These results highlight the difficulties of encouraging individuals to make lifestyle changes and the challenges in maintaining them.

## Strengths and Limitations

The tailormade HIIT intervention performed in the patients' home environment was considered a strength of this study in contrast to supervised group sessions performed on specific days and hours at a designated training facility, which would likely interfere with non-flexible working hours and transportation if the patients are not allowed to drive after stroke. The choice of no supervision and monitoring of the daily exercise intensity was, however, a limitation of the present intervention. Also, only 8 days of physical activity were monitored using accelerometers rather than the entire intervention period. We supplemented the accelerometers with use of self-reported diaries, which may be subject to recall bias and subconsciously encourage the patients to report better performance to meet own expectations or those of the researcher. In this study we failed to recruit the power-estimated predetermined number of patients due to a low recruitment rate. Since no effect of exercise was detected (*p* = 0.90), it is highly unlikely that a statistically significant difference would have been reached by inclusion of 15 more patients in each group. Recruitment bias may have occurred since patients, who were already interested in physical exercise were probably more likely to participate in a study investigating an exercise intervention. Another point to raise is the sex distribution of the sample as the majority of the included patients were male (78%), which could influence the generalisability of the findings. However, the present sample reflects the general population of patients with stroke quite well—i.e., higher incidence of stroke in younger men compared to aged-matched women (76). Also, the included patients have had a minor stroke (lacunar stroke) which limits the generalisability of the results to other categories of stroke patients.

## Conclusion

The home-based HIIT protocol in the present study was safe and well-received by the patients. The compliance was good leading to increased vigorous-intensity physical activity and training on a daily basis. This increase in physical activity was however not translated into an effect in cardiorespiratory fitness, general well-being, or improvement in biomarkers within the first 3 months of training. This may be caused by a small sample size, insufficient intensity of exercise or a bias in selection of patient who were already physically active on enrolment. Further studies investigating how this exercise approach can be optimized with special emphasis on monitoring of exercise intensity, and on finding the ideal training volume, are warranted to develop an effective HIIT protocol in patients with lacunar stroke.

## Data Availability

The datasets for this manuscript are not publicly available because: according to The Danish Data Protection Agency, a general sharing of patient data is not allowed. Requests to access the datasets should be directed to Christina Kruuse, ckruuse@dadlnet.dk.

## Ethics Statement

This study was carried out in accordance with the recommendations of the CONSORT statement, with written informed consent from all subjects. All subjects gave written informed consent in accordance with the Declaration of Helsinki. The protocol was approved by the research ethics committee in the Capital Region of Denmark.

## Author Contributions

CK and AV contributed substantial to the study conception and design, supervised and facilitated all work performed at the stroke unit, Herlev Gentofte hospital and provided key intellectual content to the manuscript. CK and RS obtained funding for the study. RS contributed substantial to the study conception and design, coordinated, planned and performed the intervention, including the patient assessments, processed data, and performed statistical work. The statistical analysis was performed in collaboration with TK and ER who also provided key content and critically reviewed the manuscript. JF and NP contributed to the study design, supervised data acquisition, and provided key content to the manuscript. HI and TC contributed to the data acquisition and provided key content to the discussion section and critically reviewed the manuscript. SR and KL contributed to the data analysis, interpretation of results, and critically reviewed the manuscript. The manuscript was drafted by RS, AV, and CK with contributions from all other authors. All authors gave final approval for the paper to be published.

### Conflict of Interest Statement

The authors declare that the research was conducted in the absence of any commercial or financial relationships that could be construed as a potential conflict of interest.

## Abbreviations

AI: Augmentation index
ANCOVA: Analysis of covariance
BMI: Body mass index
cLDA: Constrained longitudinal data analysis
CT: Computed tomography scan
GCT-TT: Graded Cycling Test with Talk Test
HIIT: High-intensity interval training
ICAM-1: Intercellular adhesion molecule-1
IL-6: Interleukin-6
IQR: Interquartile range
MDI: Major depression inventory
MET: Metabolic equivalent of task
MFI-20: Multidimensional fatigue inventory
MICE: Moderate-intensity continuous training
MoCA: Montreal cognitive assessment
MRI: Magnetic resonance imaging
PPS: Pressure pain sensitivity
Pro-ADM: Pro-adrenomedullin
Pro-ANP: Pro-atrial natriuretic peptide
RHI: Reactive hyperaemia index
SD: Standard deviation
SEM_95_: Standard error of measurement with 95 % certainty
SRD: Smallest real difference
TIA: Transient ischemic attack
TNF: Tumor necrosis factor
TOAST: Trial of Org. 10172 in acute stroke treatment
VCAM-1: Vascular cell adhesion molecule-1
VEGF: Vascular endothelial growth factor.
