# Supplementary figures and images for: Effect of Home-Based High-Intensity Interval Training in Patients With Lacunar Stroke: A Randomized Controlled Trial
Source: Front Neurol. 2019 Jun 28;10:664. doi: 10.3389/fneur.2019.00664 (PMC6611174; doi:10.3389/fneur.2019.00664)

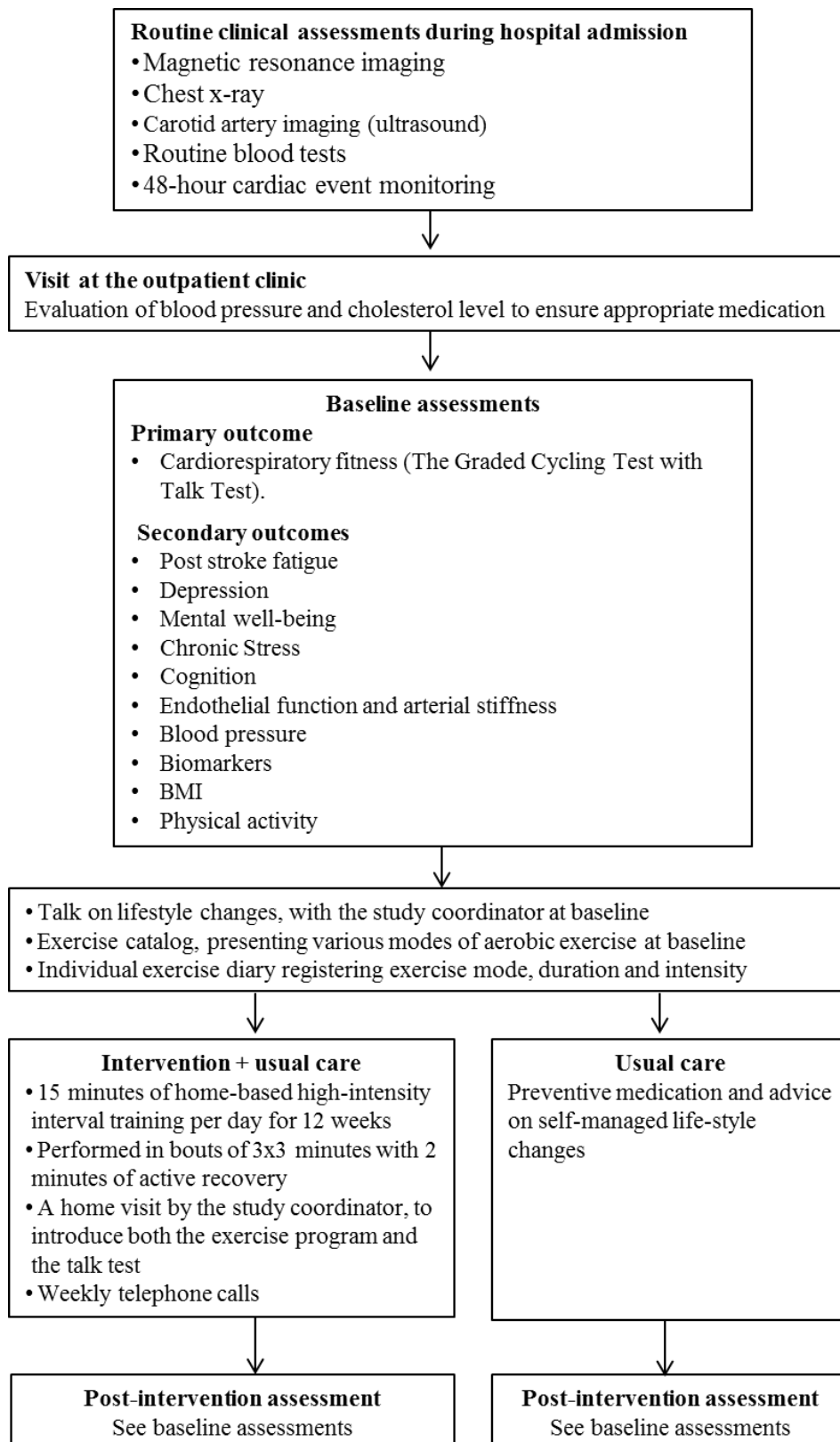

**Figure S1. Overview of study procedure**

Supplement: Supplementary file 1 [file Image_1.pdf]
