# Supplementary material for: Effect of Home-Based High-Intensity Interval Training in Patients With Lacunar Stroke: A Randomized Controlled Trial
Source: Front Neurol. 2019 Jun 28;10:664. doi: 10.3389/fneur.2019.00664 (PMC6611174; doi:10.3389/fneur.2019.00664)
Supplement: Supplementary file 2 [file Image_2.pdf]

Week no.	-2	-1	Baseline assessment	1	2	3	4	5	6	7	8	9	10	11	12	Post-intervention assessment	13
**Cardiorespiratory fitness**			X													X	
**Post-stroke fatigue**			X													X	
**Chronic stress**			X													X	
**Depression**			X													X	
**Mental well-being**			X													X	
**Cognition**			X													X	
**Endothelial function and arterial stiffness**			X													X	
**Blood pressure**			X													X	
**Biomarkers**			X													X	
**BMI**			X													X	
**AX3,** recording 8 days from assessment			→													→	
**PAS2,** questionnaire returned on assessment visit, reporting average physical activity for the past two weeks	←													←			

Figure S2. Time schedule for study assessments.
AX3: an accelerometer measuring activity patterns for everyday physical activity.
PAS2: Physical Activity Scale version 2.1 - a self-reported questionnaire of average time spent on everyday physical activity at different intensities.
